# Monochromatic image reconstruction via machine learning

**DOI:** 10.1088/2632-2153/abdbff

**Published:** 2021-04-14

**Authors:** Wenxiang Cong, Yan Xi, Bruno De Man, Ge Wang

**Affiliations:** 1 Department of Biomedical Engineering, Rensselaer Polytechnic Institute, Biomedical Imaging Center, Troy, NY 12180, United States of America; 2 Shanghai First-Imaging Tech, Shanghai, People’s Republic of China; 3 GE Research, One Research Circle, Niskayuna, NY 12309, United States of America

**Keywords:** computed tomography (CT), radon transform, monochromatic image reconstruction, machine learning

## Abstract

X-ray computed tomography (CT) is a nondestructive imaging technique to reconstruct cross-sectional images of an object using x-ray measurements taken from different view angles for medical diagnosis, therapeutic planning, security screening, and other applications. In clinical practice, the x-ray tube emits polychromatic x-rays, and the x-ray detector array operates in the energy-integrating mode to acquire energy intensity. This physical process of x-ray imaging is accurately described by an energy-dependent non-linear integral equation on the basis of the Beer–Lambert law. However, the non-linear model is not invertible using a computationally efficient solution and is often approximated as a linear integral model in the form of the Radon transform, which basically loses energy-dependent information. This approximate model produces an inaccurate quantification of attenuation images, suffering from beam-hardening effects. In this paper, a machine learning-based approach is proposed to correct the model mismatch to achieve quantitative CT imaging. Specifically, a one-dimensional network model is proposed to learn a non-linear transform from a training dataset to map a polychromatic CT image to its monochromatic sinogram at a pre-specified energy level, realizing virtual monochromatic (VM) imaging effectively and efficiently. Our results show that the proposed method recovers high-quality monochromatic projections with an average relative error of less than 2%. The resultant x-ray VM imaging can be applied for beam-hardening correction, material differentiation and tissue characterization, and proton therapy treatment planning.

## 1. Introduction

Computed tomography (CT) is a three-dimensional nondestructive imaging modality, which allows visualization and quantification of anatomical structures of human tissues with fine spatial resolution and high contrast resolution for medical diagnosis, therapeutic planning, security screening, and other applications. In clinical practice, the x-ray tube emits polychromatic x-rays, and the x-ray detector array operates in the energy-integrating mode to acquire energy intensity [1]. This physical process of x-ray imaging is accurately described by an energy-dependent non-linear integral model on the basis of the Beer–Lambert law. However, the non-linear imaging model is not invertible using a computationally efficient solution, and often approximated as a linear integral model in the form of the Radon transform, which basically loses x-ray energy-dependent information [1, 2]. Because lower energy photons are more easily attenuated in the tissues than higher energy photons, the x-ray spectral distribution at a specific location may be inconsistent for different x-ray transmission paths. The attenuation of x-rays is path-dependent and the attenuation characteristics are non-linear. Therefore, the attenuation coefficient reconstructed from the linear integral model would generate inaccurate quantification of attenuation and induce beam-hardening artifacts in the image reconstruction [3, 4].

In addition, conventional clinical CT only reveals the tissues morphology, and does not provide any information about the chemical composition of the tissues. Biological tissues are mainly composed of hydrogen, oxygen, nitrogen, and carbon. Their absorption characteristics are significantly different from that of elements with higher atomic weight, such as calcium and iodine. Iodinated contrast is often used in a medical CT exam to amplify subtle differences between tissues and visualize vasculatures, improving detectability and diagnosis of cardiac, cancer, and other diseases [5]. However, contrast-enhanced structures may have similar density to bones or calcified plaques, making them difficult to be distinguished using single-spectrum CT.

To enhance imaging performance, dual-energy computed tomography (DECT) is developed for several clinical applications. Currently, state of the art DECT scanners include fast switching of the x-ray tube voltage or kVp (General Electric’s CT750HD and Revolution CT), dual layer detectors (Philips’ IQon), and operating two beamlines at different kVp (Siemens’ dual-source CT) [6, 7]. Physically, the photon attenuation is material- and energy-dependent, and is a combined effect of photoelectric absorption and Compton scattering in the diagnostic energy range [8]. DECT acquires two projection data sets at two different energy spectra to reconstruct energy-dependent linear attenuation coefficients of the tissue, which can be used for the determination of the electron density and effective atomic number of materials, facilitating the characterization of materials and identification of tissue types [9, 10]. Using DECT techniques, material decomposition methods are developed to provide quantitative information on tissue composition to distinguish soft tissue, calcium, and iodine for important clinical applications [9, 11–13], such as urinary stone characterization, automated bone removal in CT angiography, perfused blood volume quantification. Another exemplary application of x-ray virtual monochromatic (VM) imaging is for proton therapy treatment planning. Proton therapy delivers a highly focused radiation dose at the Bragg peak, which is conformed tightly around a tumor to kill cancer cells. The stopping power ratio can be calculated from the electron density and effective atomic number of matter for the determination of Bragg peak position [14–17].

However, DECT can increase system complexity and equipment cost relative to single-spectrum CT due to the acquisitions of two spectrally different projection datasets. Emerging machine learning (ML) techniques are capable of implementing non-linear mapping, feature extraction and representation, and are widely applied for image classification, identification, super-resolution imaging, and image denoising [18–24]. In 2017, ML-based monochromatic image reconstruction method was first proposed to map polychromatic CT images to monochromatic sinogram at a pre-specified energy level based on a fully connected neural network [25]. In 2018, a deep learning method was proposed to reconstruct the VM attenuation images from multiple energy CT images using a fully connected neural network for reducing image noise and suppressing artifacts in multiple energy CT images [26]. Furthermore, a Wasserstein generative adversarial network with a hybrid loss was proposed to transform several polychromatic images with different energy bins to VM images [27]. Based on a convolutional neural network, DECT data can be also generated from single-spectrum CT data using the deep learning [28, 29]. These preliminary studies show the feasibility of x-ray VM imaging. In this paper, we propose a ML-based method to learn a non-linear transform from a training dataset to map the polychromatic CT image to a monochromatic sinogram at a pre-specified energy level. In section 2, we give a detailed description for the x-ray imaging and physical model. The one-dimensional deep network architecture is presented in detail. In section 3, based on a clinical DECT dataset, we perform the network training and testing to evaluate the proposed ML-based VM imaging. We conclude the paper in the last section.

## 2. Methodology

### 2.1. Optimization model

In medical CT, the x-ray source generally emits polychromatic x-ray photons, and the x-ray detector array operates in the energy-integrating mode to acquire energy intensity. The x-ray linear attenuation coefficient depends on both material composition of the imaged object and the x-ray photon energy. If an x-ray beam passes through an object, the x-ray transmitted beam intensity *I*(*l*) is accurately described by the non-linear integral model based on Beer–Lambert law [1, 8]:

(1) $$I(l)=\int S(E)D(E)\text{exp}\left(-\int_{l}\mu (r,E)\text{d}r\right)\text{d}E,$$

where *S*(*E*) is the energy spectrum of the x-ray source, *D*(*E*) is the detection efficiency, and *μ*(*r*, *E*) is the energy-dependent linear attenuation coefficient at an energy *E* and a spatial position *r* along a linear path *l* through the object. Using the integral mean value theorem, there is an energy level *ε*_*l*_ for the x-ray transmission path such that the following formula holds:

(2) $$\{\begin{array}{l} I(l)=I_{0}(l)\text{exp}\left(-\int_{l}\mu \left(r,\varepsilon_{l}\right)\text{d}r\right)\\ I_{0}(l)=\int_{E_{\text{min}}}^{E_{\text{max}}}S(E)D(E)\text{d}E \end{array}.$$

Equation (2) is equivalent to

(3) $$\int_{l}\mu \left(r,\varepsilon_{l}\right)\text{d}r=\text{log}\left[\frac{I_{0}(l)}{I(l)}\right],$$

where *I*_0_ (*l*) is the x-ray intensity along the path *l* without any object in the field of view. Equation (3) indicates that the line integral along a transmission path *l* relies on energy levels *ε*_*l*_. Because lower energy photons are more attenuated by tissues than higher energy photons, the x-ray spectral distribution on different x-ray transmission paths may be different. Thus, the line integral along different transmission paths may correspond to different energy levels in equation (3). Hence, the x-ray physical model described in equation (3) is different from the Radon transform without energy dependence. The mismatch of the physical model may induce beam-hardening artifacts in the image reconstruction [2].

**Figure 1.**
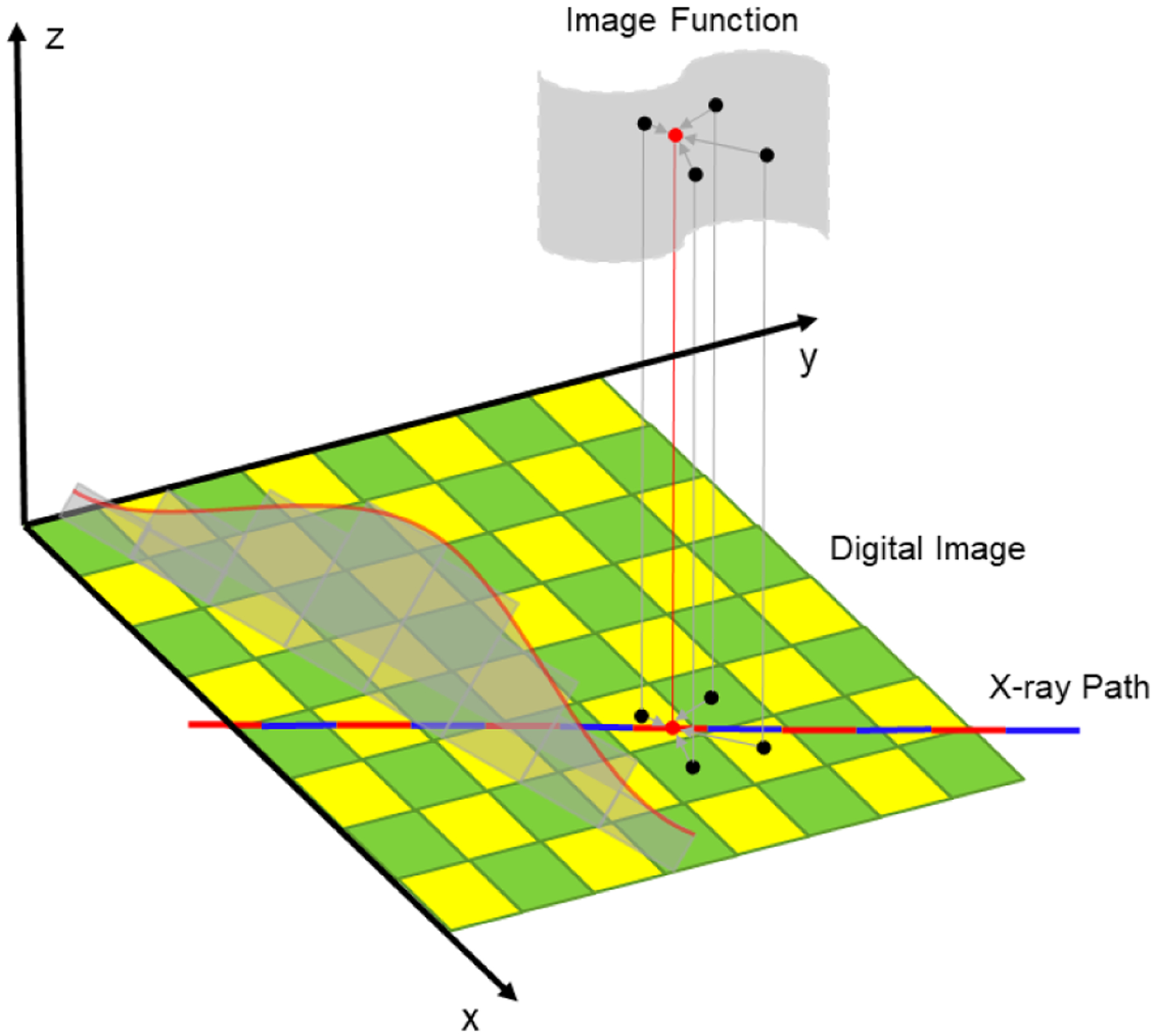
The attenuation distribution along an x-ray transmission path. Specifically, the x-ray path is first partitioned into segments of equal length. Then, the image value at the mid-point of each segment is bi-linearly interpolated from four nearest pixel values. Finally, the attenuation distribution along the x-ray transmission path is calculated by multiplying the value of each segment and the length of the segment, and processed into the corresponding line integral.

The purpose of this research is to establish a transform relation between polychromatic CT images and monochromatic sinograms at a pre-determined energy level *ε* in the detectable energy range, using a ML technique. A polychromatic attenuation image *μ*(*r*) is reconstructed from log-transformed raw data collected at all x-ray transmission paths and viewing angles. The image *μ*(*r*) contains abundant prior information on the object structure and x-ray attenuation information. A practical method is to map attenuation distribution on the x-ray transmission path *l* in the polychromatic image *μ*(*r*) to corresponding monochromatic projection datum at the specific energy level *ε*, denoted by *p*_mon_ (*l*, *ε*). This transform relation can be described mathematically by an optimization model as follows [25]:

(4) $$M=\text{argmin}\sum_{l\in \text{ all path set }}\left\|M(\mu (r),\;r\in l)-p_{\text{mon }}(l,\varepsilon )\right\|,$$

where M denotes a transformation relation, *μ*(*r*) is the polychromatic attenuation image reconstructed from a single-spectrum CT, *l* is an x-ray transmission path indexed by the location variable *r*, and *p*_mon_ (*l*, *ε*) is the monochromatic projection data at a pre-specified energy level *ε*, which can be calculated from the corresponding labeled monochromatic CT image at the energy level *ε* using the standard ray-tracing method. Specifically, we assume that all x-ray transmission paths have the same length, equal to the diameter of the field of view. The transmission path is divided into a fixed number of segments, which is denoted as *n*. Typically, *n* is chosen larger than $\sqrt{2}\times$ the input image size for sufficient sampling. The attenuation coefficient of each segment is calculated from an input polychromatic CT image using bi-linear interpolation, as shown in figure 1. The involved computation is fairly straightforward, identical to the ray-tracing process in a typical iterative reconstruction, such as algebraic reconstruction technique (ART) or simultaneous algebraic reconstruction technique (SART). As input data, the attenuation distribution along the x-ray transmission path is calculated by multiplying the attenuation coefficient of each segment by the length of the segment.

### 2.2. Architecture of a one-dimensional fully connected network with a shortcut connection

**Figure 2.**
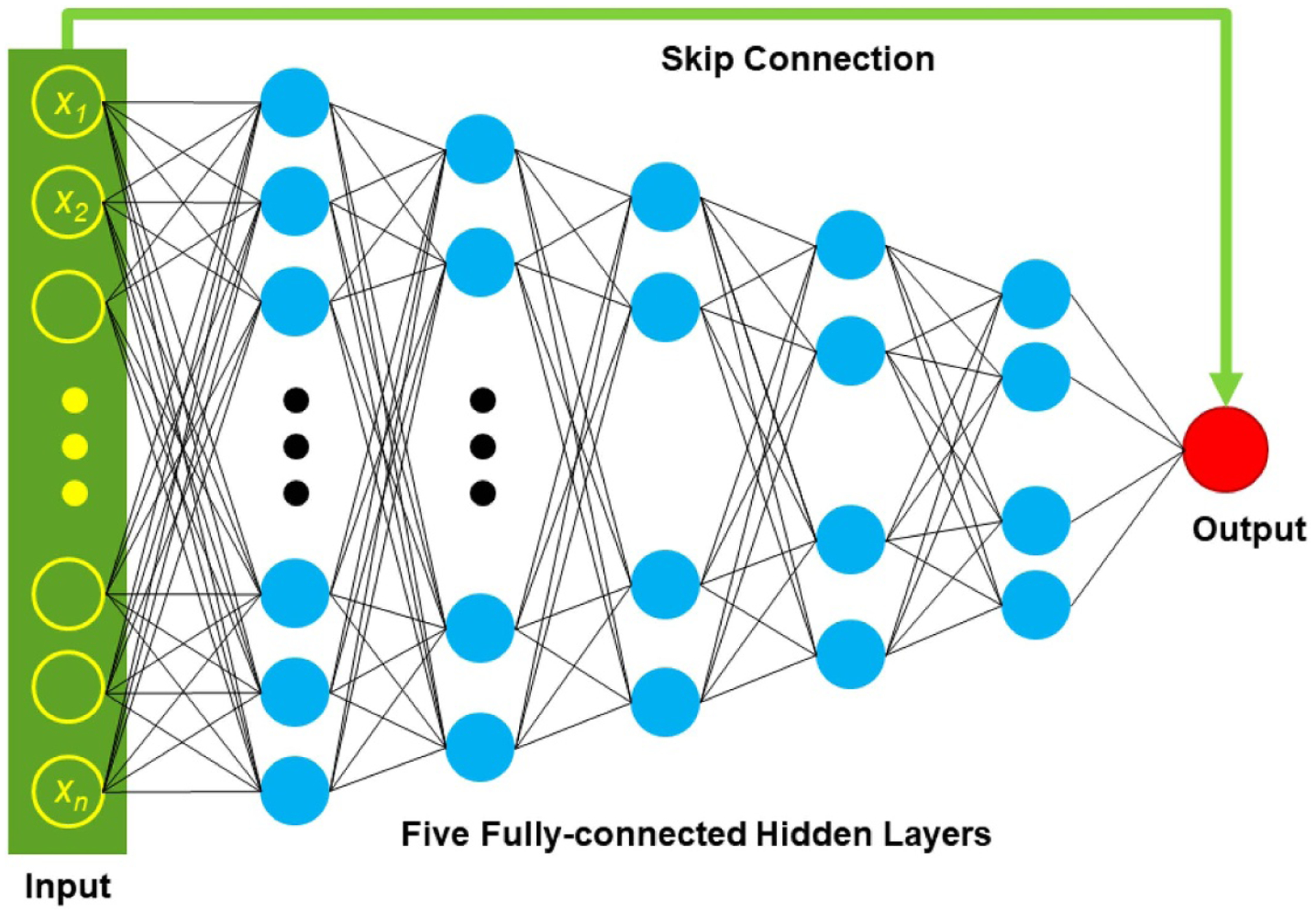
One-dimensional fully connected deep neural network with a shortcut connection.

A well-defined fully connected neural network is capable of learning any function [30]. To implement the optimization model in equation (4), we establish a one-dimensional fully connected deep network, allowing a much lower computational cost and much less memory requirement than higher dimensional networks. The neural network consists of seven layers, including an input layer, five hidden layers, an output layer, and a shortcut connection, as shown in figure 2. The first hidden layer contains 1024 neurons, the second hidden layer contains 512 neurons, the third hidden layer has 256 neurons, the fourth hidden layer contains 128 neurons, and the fifth hidden layer has 64 neurons. For every hidden layer, neurons receive the weighted combinations of neuron values on the previous layer and perform corresponding sigmoid activations. Inspired by the idea of the two-dimensional ResNet [31], the shortcut connection technique is adapted to stack the line integral of the input polychromatic image along the transmission path to the neuron on the output layer to implement a residual mapping. For implementation of the shortcut connection, a summation of the attenuation distribution along the transmission path on the input polychromatic CT image can reproduce an approximate energy-integrating projection value {log[*I*_0_ (*l*)/*I*(*l*)]}. The linear integral model is approximate in CT imaging, and usually leads to inaccurate quantification of reconstructed images. The proposed VM imaging approach is to correct the errors/mismatches of the line integral model, which can be effectively implemented by the residual mapping scheme. Prior works show that modeling residual mapping is easier than the original mapping [31]. The shortcut connection helps train neural networks efficiently. The input of the network is attenuation distribution along an x-ray transmission path *l* on the polychromatic CT image *μ*(*r*). The x-ray transmission path is divided into 1024 segments to describe the corresponding attenuation coefficient distribution. The output layer only contains a single neuron yielding a weighted linear combination of all neuron units on the last hidden layer, and outputs an x-ray monochromatic projection along the corresponding x-ray transmission path. The loss function uses the *l*_1_ norm to evaluate the difference between the predicted monochromatic projection value and the monochromatic projection value along the same transmission path for the label image.

A well-trained fully connected deep network would be a non-linear transform M that maps the polychromatic CT images to the monochromatic projection data at energy level *ε*. Once the monochromatic projections are obtained through ML, a monochromatic image *μ*(*r*, *ε*) can be reconstructed using a standard image reconstruction algorithm such as a filtered backprojection (FBP) or iterative algorithm (ART or SART) based on following line integral model:

(5) $$\int_{l}\mu (r,\varepsilon )\text{d}r=p_{\text{mon}}(l,\varepsilon ).$$

Equation (5) is an accurate x-ray imaging model at energy level *ε*. Therefore, the monochromatic CT image can be reconstructed based on equation (5), achieving accurate quantification of attenuation images and overcoming beam-hardening artifacts.

### 2.3. Ablation study

We performed an ablation study on the number of neurons per layer, the number of layers in the network, activation functions, and so on. The proposed network architecture has five hidden layers, where the number of neurons in each layer is half the number of neurons in the previous layer. This network was selected based on our extensive simulation and experimental results, some of which are described below. To demonstrate the performance of the proposed network, we also trained a multilayer perceptron (MLP) with the same number of neurons in each of the five hidden layers, and evaluated its convergence and accuracy. We found that our proposed network showed an excellent convergence and accuracy, comparable to that of the MLP, while the proposed network only requires a half the memory and half the computational cost. Especially, compared with the network architecture without a shortcut connection, the convergence speed of the network architecture with a shortcut connection was increased by more than three times. Moreover, we applied different activation functions for our neural network. The experiments show that the sigmoid is an appropriate activation function for this application. For example, if we use the ReLU activation function for all the hidden layers, the average relative error of the reconstructed monochromatic projection would exceed 20%, which is ten times higher than that of the proposed network architecture. The proposed network represents an excellent balance between accuracy, efficiency, and robustness.

## 3. Experiments and results

In clinical practice, medical x-ray imaging systems use polychromatic x-ray tubes, and the x-ray detector array operates in the energy-integrating mode to acquire energy intensity. An ideal monochromatic imaging device is not available using current x-ray sources and detectors. DECT can be used to generate VM images. In this context, VM images and polychromatic images from DECT are applied for the training and testing of the proposed neural network. In our study, the parallel-beam geometry was used for the ray-tracing process, and an image matrix of 512 × 512 pixels. Over a 360° range, 720 projections are uniformly acquired, and 729 detector elements are equidistantly distributed for each projection view.

### 3.1. Clinical dataset obtained with first CT scanner

A dataset of VM CT images produced from a GE Discovery CT750HD dual-energy scanner at Ruijin Hospital in Shanghai was used for the network training, validation, and testing. The scanning was performed with fast kVp switching between 80 kVp and 140 kVp with tube current of 260 mAs. The gantry rotation time was 0.5 s. The dataset includes 274 VM CT images at 50 keV, 60 keV, 65 keV, 70 keV, 80 keV, 90 keV, 100 keV, and 110 keV, respectively. Based on Beer–Lambert law, we first synthesized polychromatic projection data from these multi-energy images using an x-ray source spectral distribution at 120 kVp/30 mA generated by public software [32]. Then, 274 polychromatic CT images were reconstructed from polychromatic projection data using the FBP algorithm. The 274 polychromatic CT images and corresponding 274 VM images at 80 keV formed the training dataset I, while 274 polychromatic CT images and corresponding 274 VM images at 110 keV formed the training dataset II. The dataset was divided into training dataset of 200 images, validation data of 50 images, and testing data of 24 images. A total of 105 million (200 images × 720 views × 729 rays) data pairs as training data were extracted along x-ray line integral paths from 200 polychromatic CT images and corresponding 200 VM images. For the one-dimensional model, 105 million data pairs were sufficient for the network training with supervised learning.

**Figure 3.**
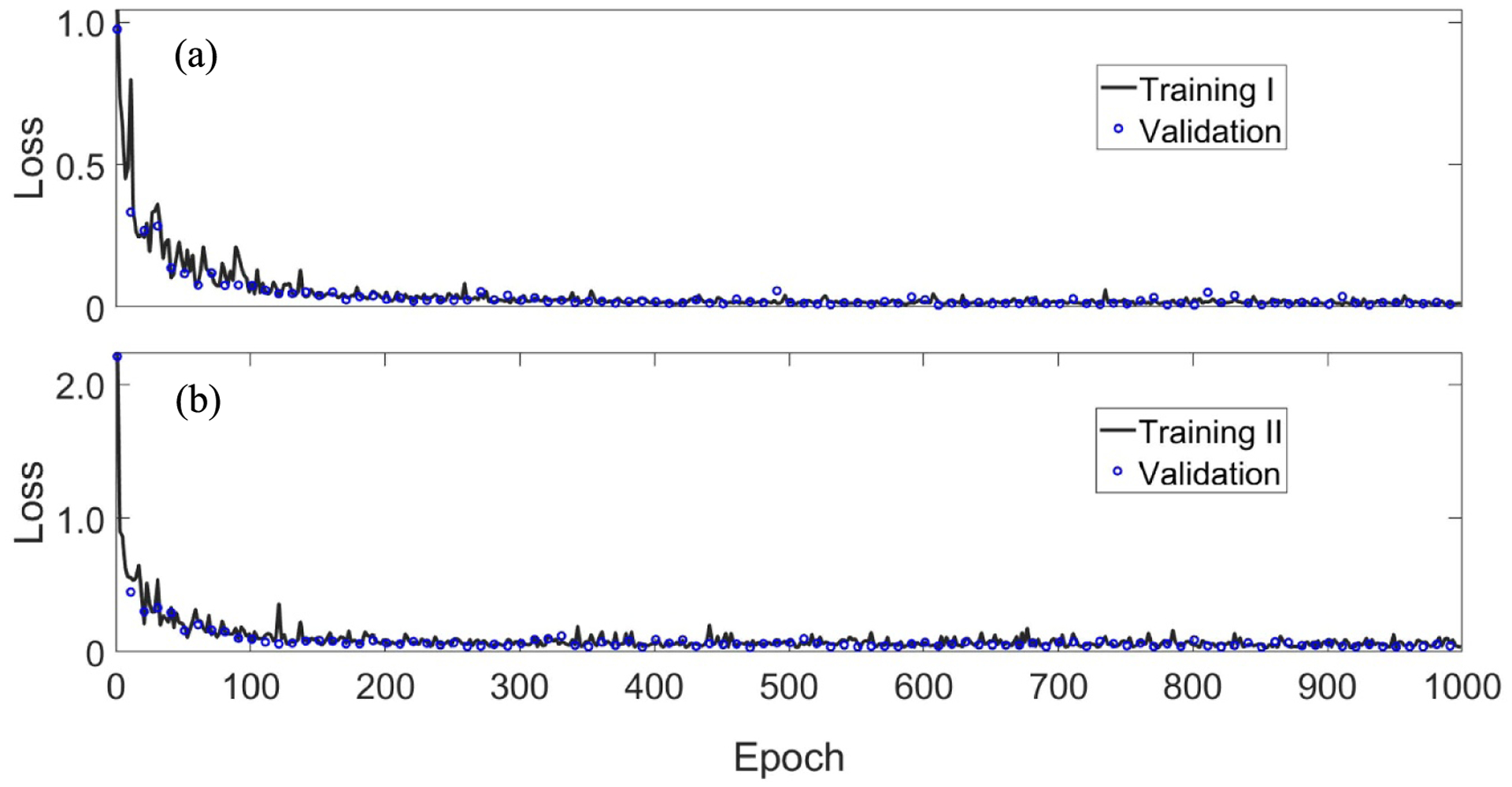
*L*_1_ norm loss versus the number of epochs. (a) and (b) are the loss function during network training I and II, respectively.

The training procedure was programmed in Python and Tensorflow on a PC with a NVIDIA Titan XP GPU with 12 GB memory. The network training was conducted using the ADAM optimization algorithm with *β*_1_ = 0.9, *β*_2_ = 0.999, epsilon = 1.0 × 10^*−*7^, and weight decay = 0.0. The learning rate was set to 10^*−*3^. Weight and bias parameters of the network were initialized randomly. Data were randomly sampled in the training dataset, maximizing the probability of finding the global minimum. Based on dataset I, the network is trained to generate monochromatic sinogram at 80 keV, while the network is trained using dataset II to generate monochromatic sinogram at 110 keV. The networks were trained in 1000 epochs within 12 h. Figure 3(a) shows the loss function during the network training using dataset I. Figure 3(b) shows the loss function during the network training using dataset II. The training of the fully connected deep network showed an excellent convergence behavior.

**Figure 4.**
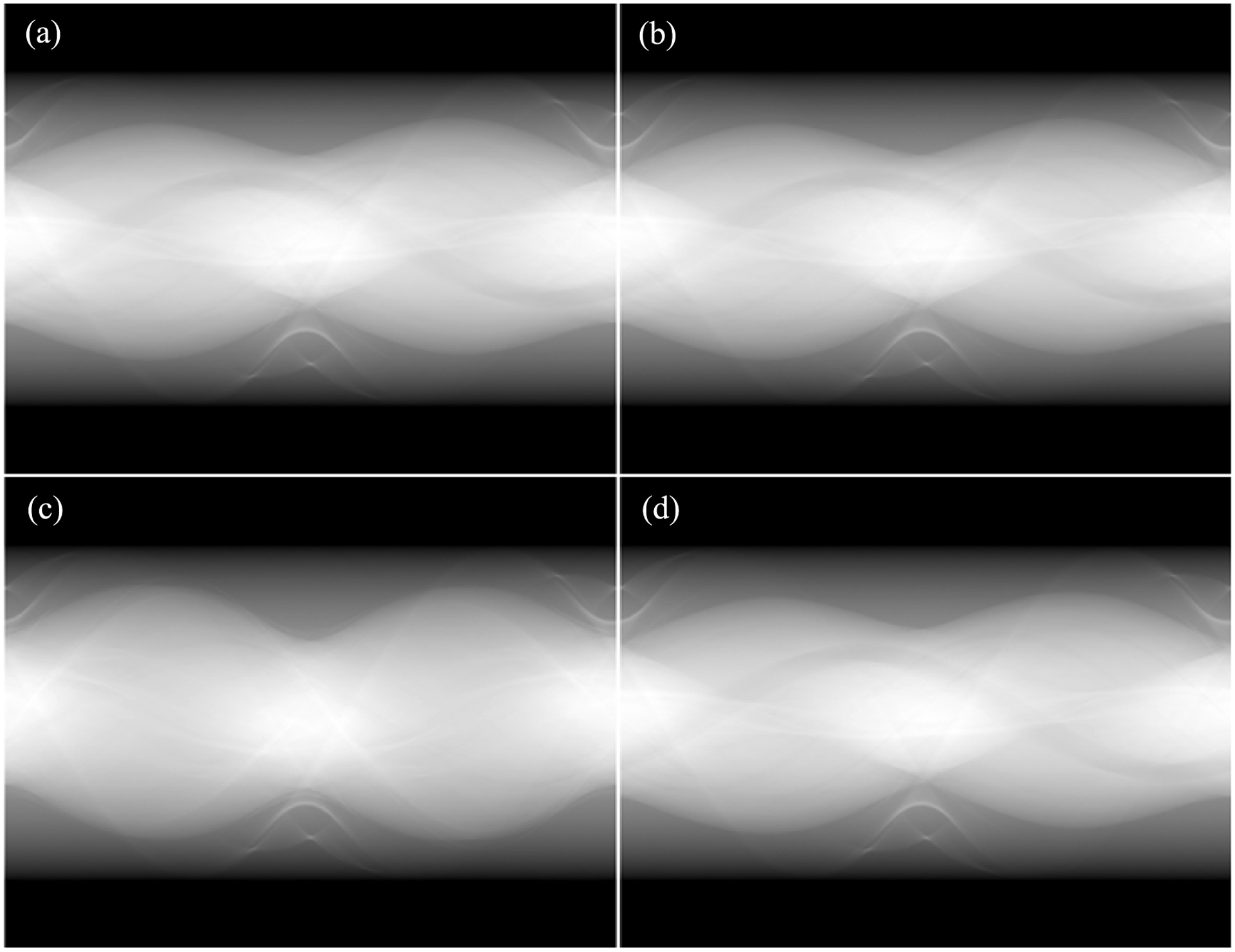
Monochromatic sinogram comparison: the estimated monochromatic sinograms at 80 keV (a) and 110 keV (c) and the label monochromatic sinograms at 80 keV (b) and 110 keV (d).

**Figure 5.**
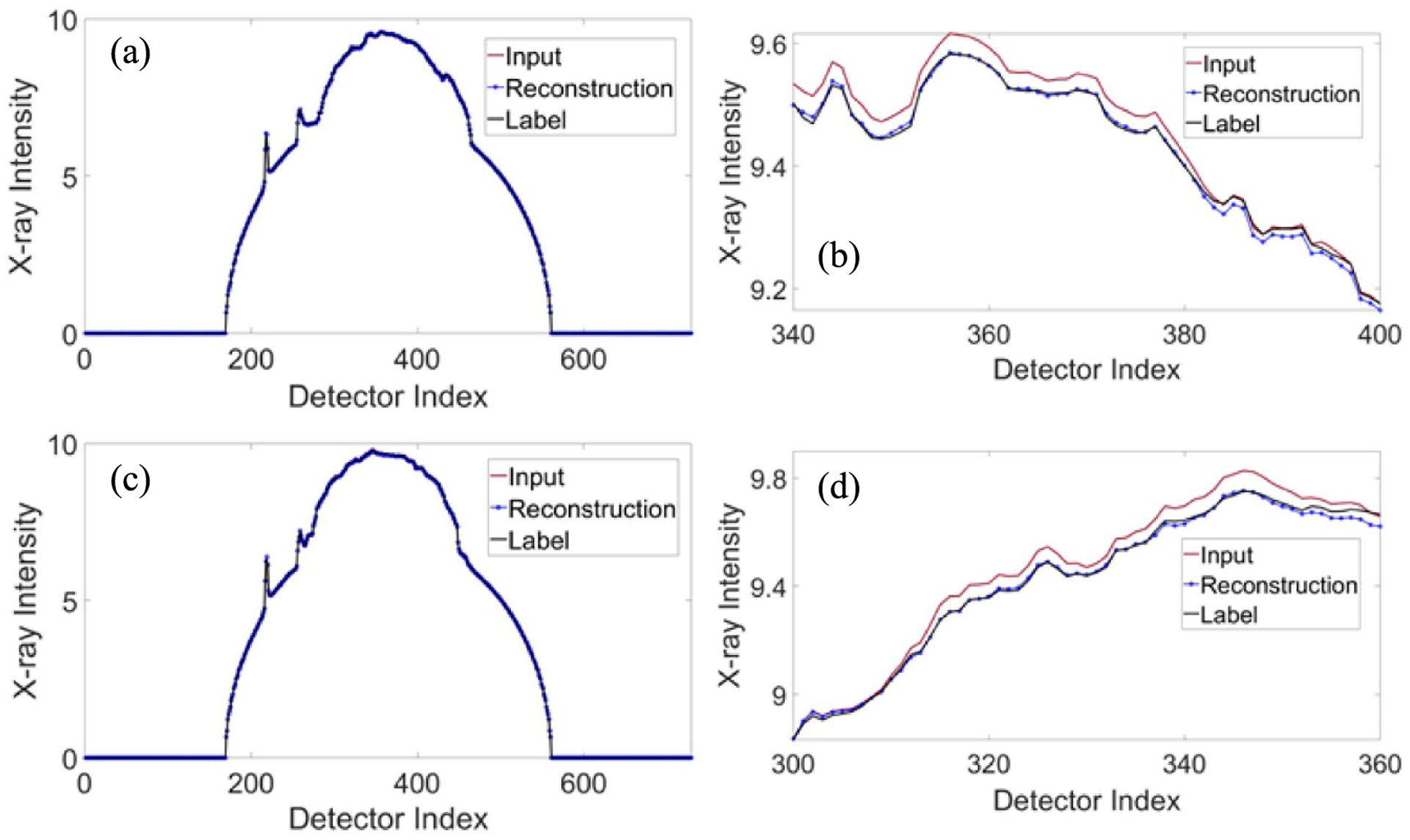
Comparison between the polychromatic x-ray projection at 120 kVp, the estimated monochromatic projection, and the corresponding label monochromatic projection: (a) the horizontal view at 80 keV; (b) a zoomed portion of the 80 keV profile; (c) the horizontal view at 110 keV; and (d) a zoomed portion of the 110 keV profile.

For the testing of the trained network, 24 polychromatic images at 120 kVp were input to the trained neural network models I and II to generate monochromatic sinogram at 80 keV and 110 keV, respectively. CT image were randomly selected in testing dataset as examples to present the quality of monochromatic imaging. The labeled monochromatic sinogram data were obtained from the labeled monochromatic CT image using the ray-tracing method. Figure 4 presents the comparison between the estimated monochromatic sinograms and the corresponding (ground-truth) labeled monochromatic sinograms at 80 keV and 110 keV respectively. Figure 5 presents the comparison between the polychromatic x-ray projection at 120 kVp, the estimated monochromatic projection, and the corresponding labeled monochromatic projection at the horizontal views. The trained neural network delivered high-quality monochromatic projection data in the testing phase, with an average relative error of less than 2%. With VM images reconstructed from DECT as the reference, we used the popular signal-to-noise ratio (PSNR) and structural similarity (SSIM) indices to evaluate the estimated monochromatic sinogram against the labeled monochromatic sinogram. The average PSNR measures are 37.54 dB and 36.89 dB for monochromatic sinograms at 80 keV and 110 keV respectively, and the average SSIM values are 0.9941 and 0.9902 for monochromatic sinograms at 80 keV and 110 keV respectively.

**Figure 6.**
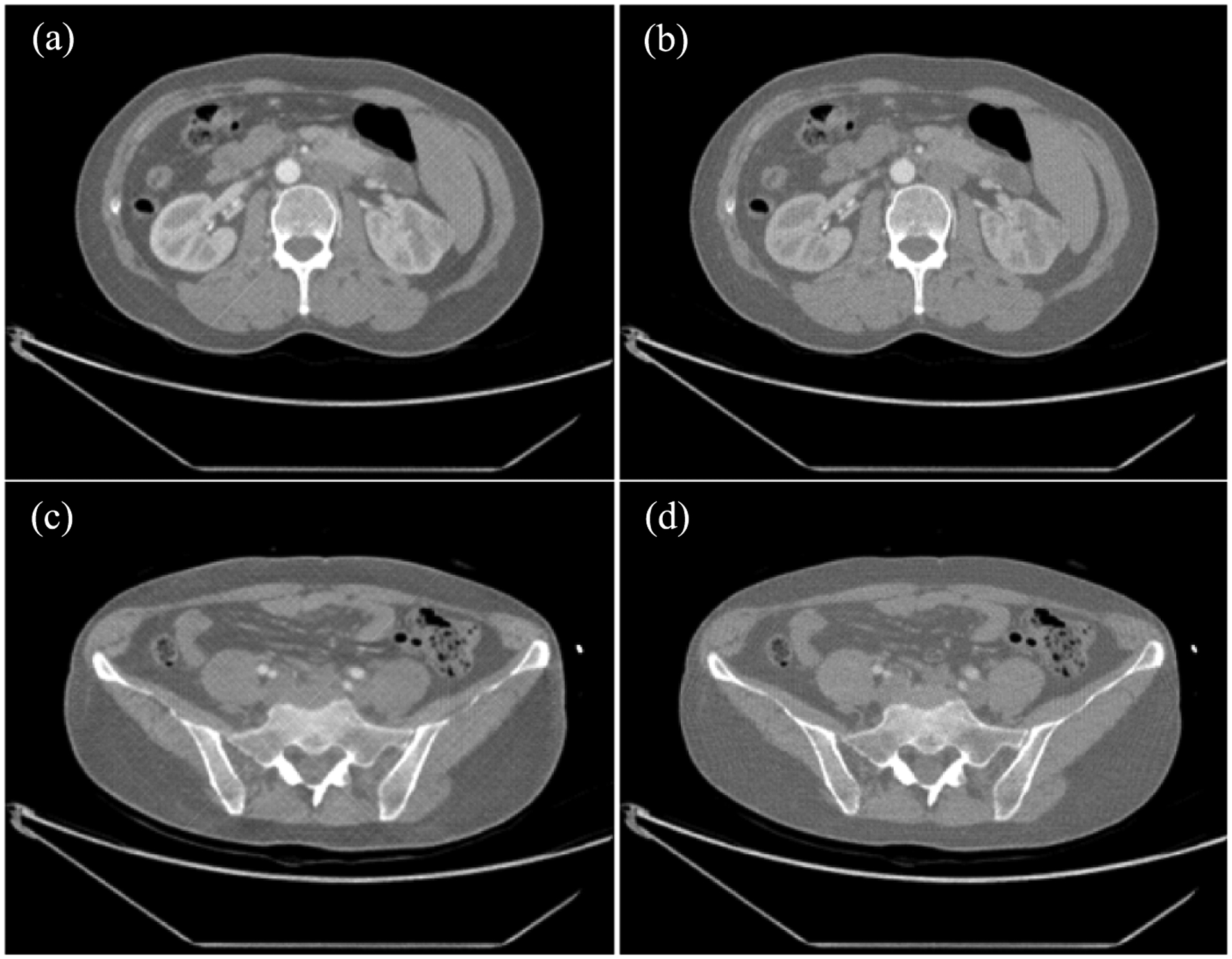
Monochromatic image reconstruction, trained based on VM CT image data. (a) VM image at 80 keV reconstructed by the deep learning-based estimation and (b) the label VM image at 80 keV reconstructed from DECT VM data. (c) VM image at 110 keV reconstructed by the deep learning-based estimation and (d) the label VM image at 110 keV reconstructed from DECT VM data. The images are displayed with a window width of 436 HU and a level of 416 HU.

Furthermore, the VM CT images at 80 keV and 110 keV were reconstructed from the estimated monochromatic sinogram at 80 keV and 110 keV using the FBP algorithm. Figure 6 presents the comparison between the estimated VM image and the label VM image at 80 keV and 110 keV. We calculated PSNR and SSIM indices to evaluate the estimated VM images at 80 keV and 110 keV. The average PSNR was 69.79 dB and 63.34 dB for the VM images at 80 keV and 110 keV, respectively, while the average SSIM was 0.9999 and 0.997 for the VM images at 80 keV and 110 keV, respectively. The proposed ML-based method well preserved structural information especially texture features and gave superior image quality.

### 3.2. Clinical dataset obtained with second CT scanner

We obtained a second series of clinical DECT dataset to further evaluate the performance of the deep learning-based VM imaging method. We now used the originally reconstructed kVp images as the input to the network, instead of the synthesized kVp images used in the previous section. DECT data of eight patients (3182 slices in total) were collected on a SOMATOM Definition Flash DECT scanner (Siemens Healthineers, Forchheim, Germany) at Ruijin Hospital in Shanghai, China. The DECT scanner worked in the dual-source scanning mode, which operated at 100 kVp/210 mAs and 140 kVp/162 mAs with a wedge filter and a flat filter respectively. The scanning was set for exposure time 0.5 s. CT images were reconstructed using the FBP algorithm. The dataset includes 3182 polychromatic CT images at 140 kVp, being associated with 3182 VM CT images at 80 keV and 3182 VM CT images at 110 keV. The dataset was split into training, validation and testing sets, which respectively came from five, two and one patients. In other words, 2195 polychromatic CT images at 140 kVp and the corresponding 2195 monochromatic images at 80 keV formed training dataset I, while 2195 polychromatic CT images at 140 kVp and the corresponding 2195 monochromatic images at 110 keV formed training dataset II. In total, 1152 million (2195 images × 720 views × 729 rays) data pairs were extracted along x-ray line integral paths from the 2195 polychromatic CT images at 140 kVp and corresponding 2195 monochromatic images in the datasets I and II for training the network models I and II respectively.

**Figure 7.**
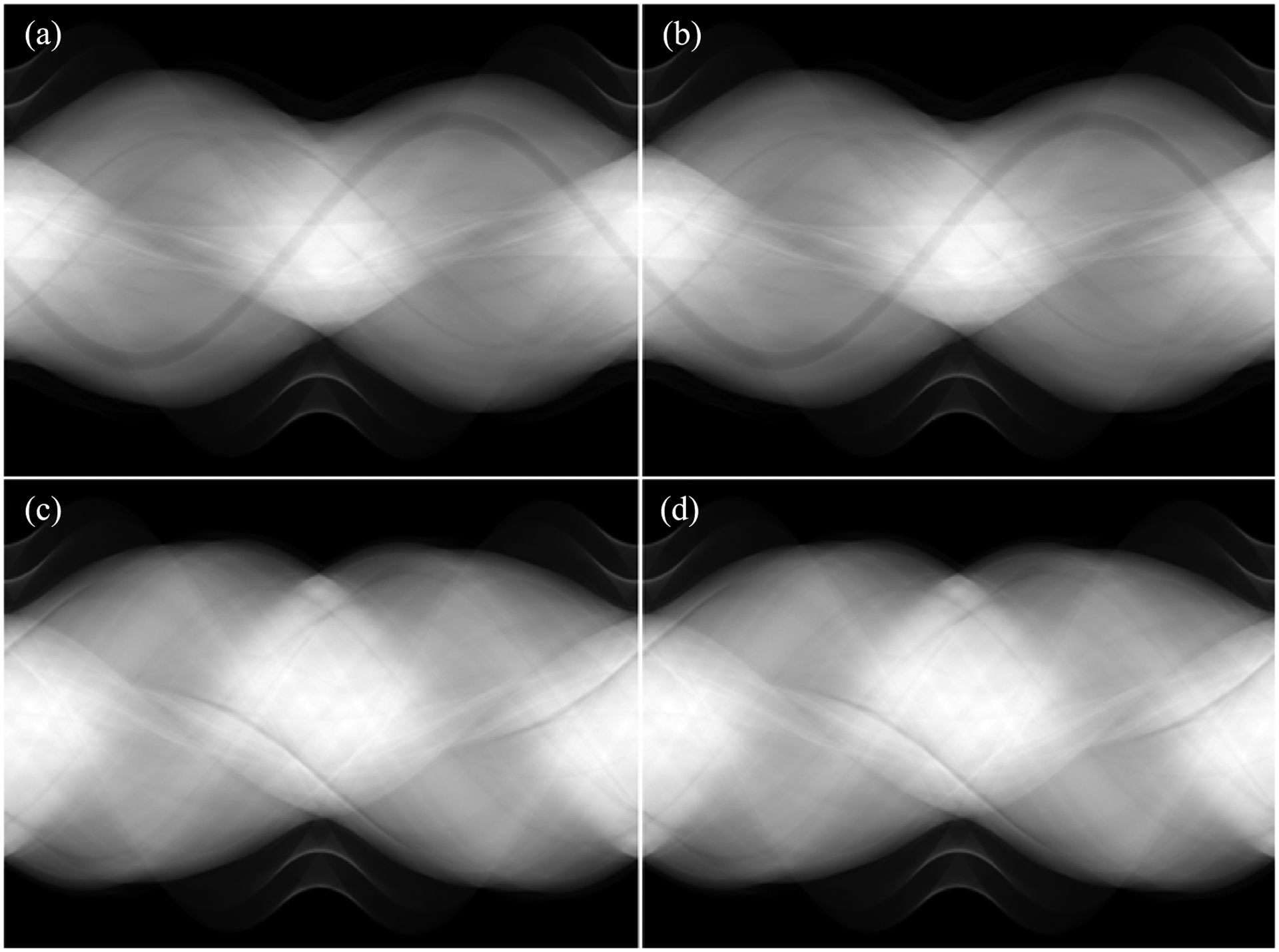
Monochromatic sinogram estimation based on kVp images. The estimated monochromatic sinograms at 80 keV (a) and 110 keV (c). The label monochromatic sinograms at 80 keV (b) and 110 keV (d).

**Figure 8.**
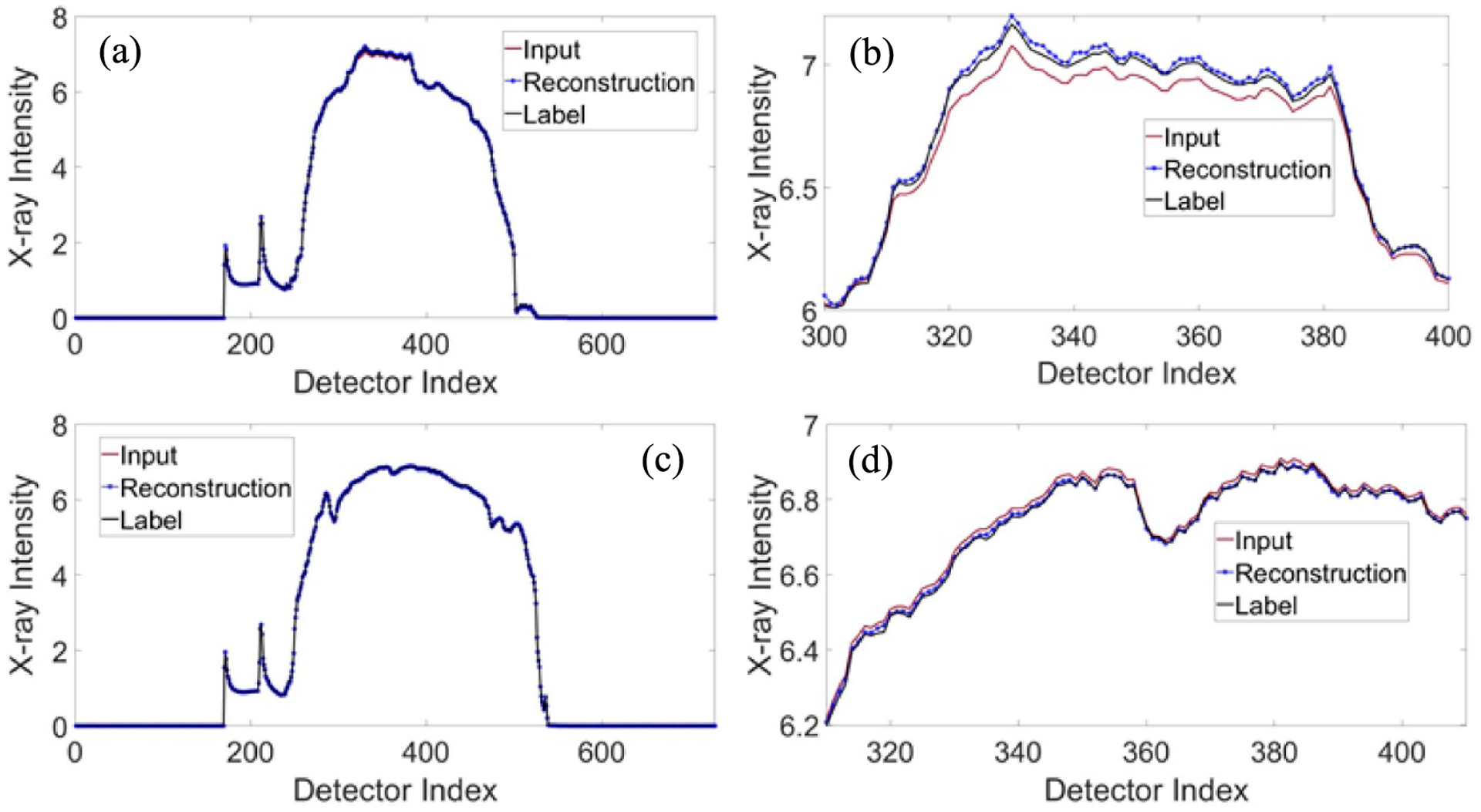
Comparison between the polychromatic x-ray projection at 140 kVp, the estimated monochromatic projection, and corresponding label monochromatic projection. (a) The horizontal view at 80 keV and (b) a zoomed portion of the 80 keV profile. (c) The horizontal view at 110 keV and (d) a zoomed portion of the 110 keV profile.

Polychromatic CT images at 140 kVp for the test datasets were input to the trained neural network models I and II to generate monochromatic sinograms at 80 keV and 110 keV, respectively. Figure 7 presents the comparison between the estimated monochromatic sinograms and the corresponding label monochromatic sinograms at 80 keV and 110 keV respectively. Figure 8 presents the comparison between the polychromatic x-ray projection at 140 kVp, the estimated monochromatic projection, and corresponding label monochromatic projection in the horizontal views. We calculated PSNR and SSIM to evaluate the estimated monochromatic sinograms with label monochromatic sinograms as reference. The average PSNR was 33.40 dB and 33.27 dB for the estimated monochromatic sinograms at 80 keV and 110 keV, respectively, while average SSIM was 0.9995 and 0.9978 for the estimated monochromatic sinograms at 80 keV and 110 keV, respectively.

**Figure 9.**
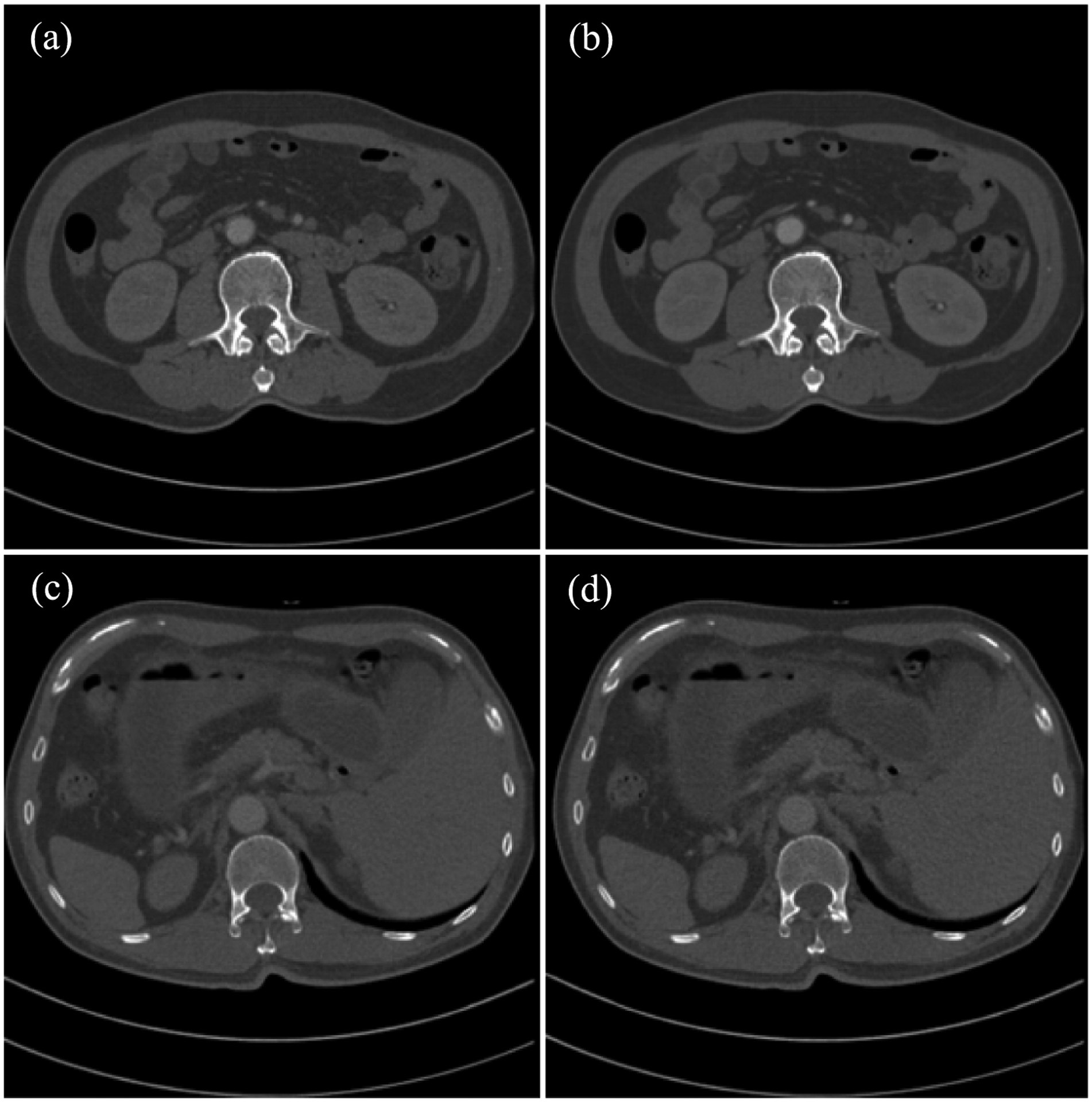
VM image reconstruction trained based on DECT data. (a) VM image at 80 keV reconstructed by the deep learning-based estimation and (b) the label VM image at 80 keV reconstructed from DECT. (c) VM image at 110 keV reconstructed by the deep learning-based estimation and (d) the label VM image at 110 keV reconstructed from DECT. The images are displayed with a window width of 981 HU and a level of 143 HU.

Again, VM images at 80 keV and 110 keV were reconstructed from the estimated monochromatic sinogram data at 80 keV and 110 keV respectively using the FBP algorithm. Figure 9 shows the comparison between the estimated VM image and the label VM images at 80 keV and 110 keV, respectively. The proposed estimation method well preserved structural information, in particular texture features, yielding a superior image quality. We calculated the PSNR and SSIM measures to evaluate the reconstructed VM images against the label VM images. The average PSNR was 48.92 dB and 48.73 dB for the reconstructed VM images at 80 keV and 110 keV respectively, while the average SSIM was 0.9940 and 0.9933 for the reconstructed VM images at 80 keV and 110 keV, respectively. Comparing image quality in terms of SSIM and PSNR in sections 3.1 and 3.2, the quality of the VM image reconstructed from real kVp images is slightly compromised relative to the quality of the VM image reconstructed from synthesized kVp images, due to non-ideal noise and spectral distortion in clinical data.

## 4. Discussions and conclusion

DECT acquires two spectrally different projection datasets for VM imaging, and can perform the characterization of materials and identification of tissue types. However, DECT suffers from increased system complexity and higher cost compared to a conventional single-spectrum CT scanner. This proposed ML-based method learns a non-linear transform from the training dataset to map polychromatic CT images to monochromatic sinogram through a powerful neural network. Unlike DECT image reconstruction from two spectrally different projection datasets, the ML-based monochromatic imaging method only utilizes a single-spectrum energy-integrating projection dataset. Our experimental results show that the neural network model has an excellent convergent behavior in the training process, and recovers high-quality monochromatic sinogram with an average relative error of less than 2%, realizing VM imaging and overcoming beam-hardening effectively and efficiently. The proposed one-dimensional network model has a much lower computational cost and a much lower memory requirement than higher dimensional counterparts.

As an x-ray beam passes through biological tissue, interactions mainly involve the photoelectric effect and Compton scattering. Photoelectric absorption occurs when an incident x-ray photon collides with an inner-shell electron in an atom, while Compton scattering is the result of the interaction between an x-ray photon and an outer orbital electron. As a result, photoelectric absorption is related to the atomic number of the attenuating medium (Z), and Compton Effect is dependent on the electron density in the absorbing material. With our proposed ML-based VM imaging, VM CT images at two energy levels can be reconstructed, and the electron density and effective atomic number of matter composition can be extracted from energy-dependent linear attenuation coefficients for material decomposition, tissue characterization, beam-hardening correction, and proton therapy planning [33].

Moreover, the proposed method is able to reconstruct energy-dependent attenuation images. So, the trained neural network model relies on the spectral distribution of x-ray source in CT scanner. The trained network model should be used to process CT images obtained from a CT scanner whose x-ray tube energy spectral distribution is similar to that of the x-ray tube generating input CT images in the training dataset.

The proposed method can be interpreted as a learning-based, advanced beam-hardening correction, since a beam-hardening correction also maps a polychromatic dataset onto a VM dataset at a given energy. It is possible that the proposed learning-based method learns to generate monochromatic datasets more accurately than conventional single-material beam-hardening correction methods, and more robustly than multi-material beam-hardening correction methods. The proposed approach is able to take into account contextual information to perform the best possible estimation. A comparison of the proposed approach relative to traditional single-material and multi-material beam-hardening correction approaches is out of scope for this paper but will be a critical focus of future work.

## Data Availability

No new data were created or analyzed in this study.

## References

[1] Buzug TM 2008 Computed Tomography: From Photon Statistics to Modern Cone-beam CT (Berlin: Springer)

[2] Hsieh J 2009 Computed Tomography Principles, Design, Artifacts, and Recent Advances (New York: Wiley)

[3] de Man B, Nuyts J, Dupont P, Marchal G and Suetens P 2001 An iterative maximum-likelihood polychromatic algorithm for CT IEEE Trans. Med. Imaging 20 999–1008 11686446 10.1109/42.959297

[4] Herman GT 1979 Correction for beam hardening in computed tomography Phys. Med. Biol 24 81–106 432276 10.1088/0031-9155/24/1/008

[5] Gupta R, Phan CM, Leidecker C, Brady TJ, Hirsch JA, Nogueira RG and Yoo AJ 2010 Evaluation of dual-energy CT for differentiating intracerebral hemorrhage from iodinated contrast material staining Radiology 257 205–11 20679449 10.1148/radiol.10091806

[6] Forghani R, de Man B and Gupta R 2017 Dual-energy computed tomography: physical principles, approaches to scanning, usage, and implementation: part 1 Neuroimaging Clin. North Am 27 371–84 10.1016/j.nic.2017.03.002 28711199

[7] Forghani R, de Man B and Gupta R 2017 Dual-energy computed tomography: physical principles, approaches to scanning, usage, and implementation: part 2 Neuroimaging Clin. North Am 27 385–400 10.1016/j.nic.2017.03.003 28711200

[8] Alvarez RE and Macovski A 1976 Energy-selective reconstructions in x-ray computerized tomography Phys. Med. Biol 21 733–44 967922 10.1088/0031-9155/21/5/002

[9] Patino M, Prochowski A, Agrawal MD, Simeone FJ, Gupta R, Hahn PF and Sahani DV 2016 Material separation using dual-energy CT: current and emerging applications Radiographics 36 1087–105 27399237 10.1148/rg.2016150220

[10] Heckert M, Enghardt S and Bauch J 2020 Novel multi-energy x-ray imaging methods: experimental results of new image processing techniques to improve material separation in computed tomography and direct radiography PLoS One 15 e0232403 32374774 10.1371/journal.pone.0232403 PMC7202619

[11] Liu X, Yu L, Primak AN and McCollough CH 2009 Quantitative imaging of element composition and mass fraction using dual-energy CT: three-material decomposition Med. Phys 36 1602–9 19544776 10.1118/1.3097632 PMC2719492

[12] Maass C, Baer M and Kachelriess M 2009 Image-based dual energy CT using optimized precorrection functions: a practical new approach of material decomposition in image domain Med. Phys 36 3818–29 19746815 10.1118/1.3157235

[13] Kayama R, Fukuda T, Ogiwara S, Momose M, Tokashiki T, Umezawa Y, Asahina A and Fukuda K 2020 Quantitative analysis of therapeutic response in psoriatic arthritis of digital joints with dual-energy CT iodine maps Sci. Rep 10 1225 31988331 10.1038/s41598-020-58235-9 PMC6985244

[14] Delaney TF and Kooy HM 2008 Proton and Charged Particle Radiotherapy (Baltimore, MD: Williams & Wilkins)

[15] Paganetti H 2011 Proton Therapy Physics (London: Taylor and Francis Group)

[16] Das IJ and Paganetti H 2015 Principles and Practice of Proton Beam Therapy (Madison, WI: Medical Physics Publishing)

[17] Newhauser WD and Zhang R 2015 The physics of proton therapy Phys. Med. Biol 60 R155–209 25803097 10.1088/0031-9155/60/8/R155 PMC4407514

[18] Danaee P, Ghaeini R and Hendrix DA 2017 A deep learning approach for cancer detection and relevant gene identification Pacific Symp. on Biocomputing vol 22 pp 219–29 10.1142/9789813207813_0022 PMC5177447 27896977

[19] Wang G 2016 A perspective on deep imaging IEEE Access 4 8914–24

[20] Nie D, Trullo R, Lian J, Wang L, Petitjean C, Ruan S, Wang Q and Shen D 2018 Medical image synthesis with deep convolutional adversarial networks IEEE Trans. Biomed. Eng 65 2720–30 29993445 10.1109/TBME.2018.2814538 PMC6398343

[21] Tang P, Wang X, Feng B and Liu W 2017 Learning multi-instance deep discriminative patterns for image classification IEEE Trans. Image Process 26 3385–96 28026762 10.1109/TIP.2016.2642781

[22] Park J, Hwang D, Kim KY, Kang SK, Kim YK and Lee JS 2018 Computed tomography super-resolution using deep convolutional neural network Phys. Med. Biol 63 145011 29923839 10.1088/1361-6560/aacdd4

[23] Shan H, Padole A, Homayounieh F, Kruger U, Khera RD, Nitiwarangkul C, Kalra MK and Wang G 2019 Competitive performance of a modularized deep neural network compared to commercial algorithms for low-dose CT image reconstruction Nat. Mach. Intell 1 269–76 33244514 10.1038/s42256-019-0057-9 PMC7687920

[24] You C 2020 CT super-resolution GAN constrained by the identical, residual, and cycle learning ensemble (GAN-CIRCLE) IEEE Trans. Med. Imaging 39 188–203 31217097 10.1109/TMI.2019.2922960 PMC11662229

[25] Cong W and Wang G 2017 Monochromatic image reconstruction from a single-spectrum CT via machine learning (arXiv:1710.03784)

[26] Feng C, Kang K and Xing Y 2019 Fully connected neural network for virtual monochromatic imaging in spectral computed tomography J. Med. Imaging 6 011006 10.1117/1.JMI.6.1.011006 PMC6197866 30397632

[27] Shi Z, Li J, Li H, Hu Q and Cao Q 2019 A virtual monochromatic imaging method for spectral CT based on Wasserstein generative adversarial network with a hybrid loss IEEE Access 7 110992–1011

[28] Zhao W, Lvu T, Gao P, Shen L, Dai X, Cheng K, Jia M, Chen Y and Xing L 2019 Dual-energy CT imaging using a single-energy CT data is feasible via deep learning (arXiv:1906.04874)

[29] Lvu T, Wu Z, Zhang Y, Chen Y, Xing L and Zhao W 2020 Dual-energy CT imaging from single-energy CT data with material decomposition convolutional neural network (arXiv:2006.00149) 10.1016/j.media.2021.102001 33640721

[30] Ramsundar B and Zadeh RB 2018 TensorFlow for Deep Learning: From Linear Regression to Reinforcement Learning 1st edn (Sebastopol, CA: O’Reilly Media)

[31] He K, Zhang X, Ren S and Sun J 2016 Deep residual learning for image recognition IEEE Conf. on Computer Vision and Pattern Recognition (CVPR) pp 770–8

[32] (Available at: https://health.siemens.com/booneweb/index.html)

[33] Bharati A, Mandal SR, Gupta AK, Seth A, Sharma R, Bhalla AS, Das CJ, Chatterjee S and Kumar P 2019 Development of a method to determine electron density and effective atomic number of high atomic number solid materials using dual-energy computed tomography J. Med. Phys 44 49–56 30983771 10.4103/jmp.JMP_125_18 PMC6438052

